# Case Report: Atypical presentation of non-functional gonadotropinoma

**DOI:** 10.12688/f1000research.133438.1

**Published:** 2023-06-14

**Authors:** Suresh Oommen, Sam Rice

**Affiliations:** 1 Endocrinology & Diabetes Department, Bronglais Hospital, NHS Wales Hywel Dda University Health Board, Aberystwyth, Wales, SY231ER, UK; 2 Endocrinology & diabetes Department, Swansea University, Swansea, Wales, UK

**Keywords:** Non-functional pituitary adenoma, gonadotropinoma, hemianopia

## Abstract

Gonadotropinoma is the most common non-functional pituitary adenoma comprising 10%–30% of all pituitary adenomas. They are benign slow-growing tumours originating from adenohypophysis and rarely become malignant. Its presentation can be atypical, such as visual disturbance, and most patients presenting to an ophthalmologist for visual correction are eventually found to have a field defect. Here, we report a case of a 59-year-old patient who presented with a left-sided visual disturbance, which progressed over the years due to a left temporal hemianopia. The patient was referred to us by an ophthalmologist and was diagnosed with a giant non-functional gonadotropinoma. The patient was surgically treated. Postoperative follow-up magnetic resonance imaging after 3 months showed near complete resection of the tumour.

## Introduction

Non-functional gonadotropinomas have become increasingly common as part of non-functional pituitary adenoma with estimated prevalence of 78–94 cases per 1,000,000 population and may present with fewer symptoms such as loss of libido, lethargy or visual disturbance because of their slow-growing nature. ^1^ We present, with informed consent, a case report of a patient who had blurring of vision in the left eye and initially presented to an ophthalmologist who made a diagnosis of left temporal hemianopia. The patient was referred to us for urgent magnetic resonance imaging (MRI), which showed a large pituitary mass compressing the optic chiasm with an anterior pituitary hormone profile showing suppressed levels of thyroxine (T4), follicle-stimulating hormone (FSH), luteinizing hormone (LH), and testosterone and mildly elevated levels of prolactin. Immunohistochemistry was positive for steroidogenic (transcription) factor 1 (SF1), indicating silent gonadotropinoma. This article is reported in line with CARE guidelines. ^11^

## Case report

A 59-year-old Welsh male, self-employed, was referred to us by an ophthalmologist with a left visual disturbance, which worsened since 2019. He complained of blurring in his left peripheral vision and was diagnosed with left temporal hemianopia, confirmed by Humphrey’s perimetry. The patient did not complain of associated headaches, nausea, vomiting upper limb weakness, cold intolerance, or weight gain. He complained of low energy and decreased libido for approximately 10 years, but showed normal development of secondary sexual characteristics and had a normal frequency of shaving. He had no cushingoid, acromegaly, or other features suggestive of multiple endocrine neoplasia syndrome. He had no significant past medical, family or psychosocial history. He was not on any medications at the time of presentation.

On examination, the patient was overweight, with a height of 175 cm and weight of 85 kg with BMI of 27. His blood pressure was 140/70 mm Hg, with a pulse of 80 bpm. His body temperature was 36.5 °C. His Glasgow coma scale score was 15/15, and eye examination showed right and left eye visual acuity of 6/6 and 6/24, respectively, as well as reduced colour vision and temporal field defect in the left eye with normal fundus examination. Respiratory, cardiac, abdominal and neurological examination findings were normal.

**Figure 1.**
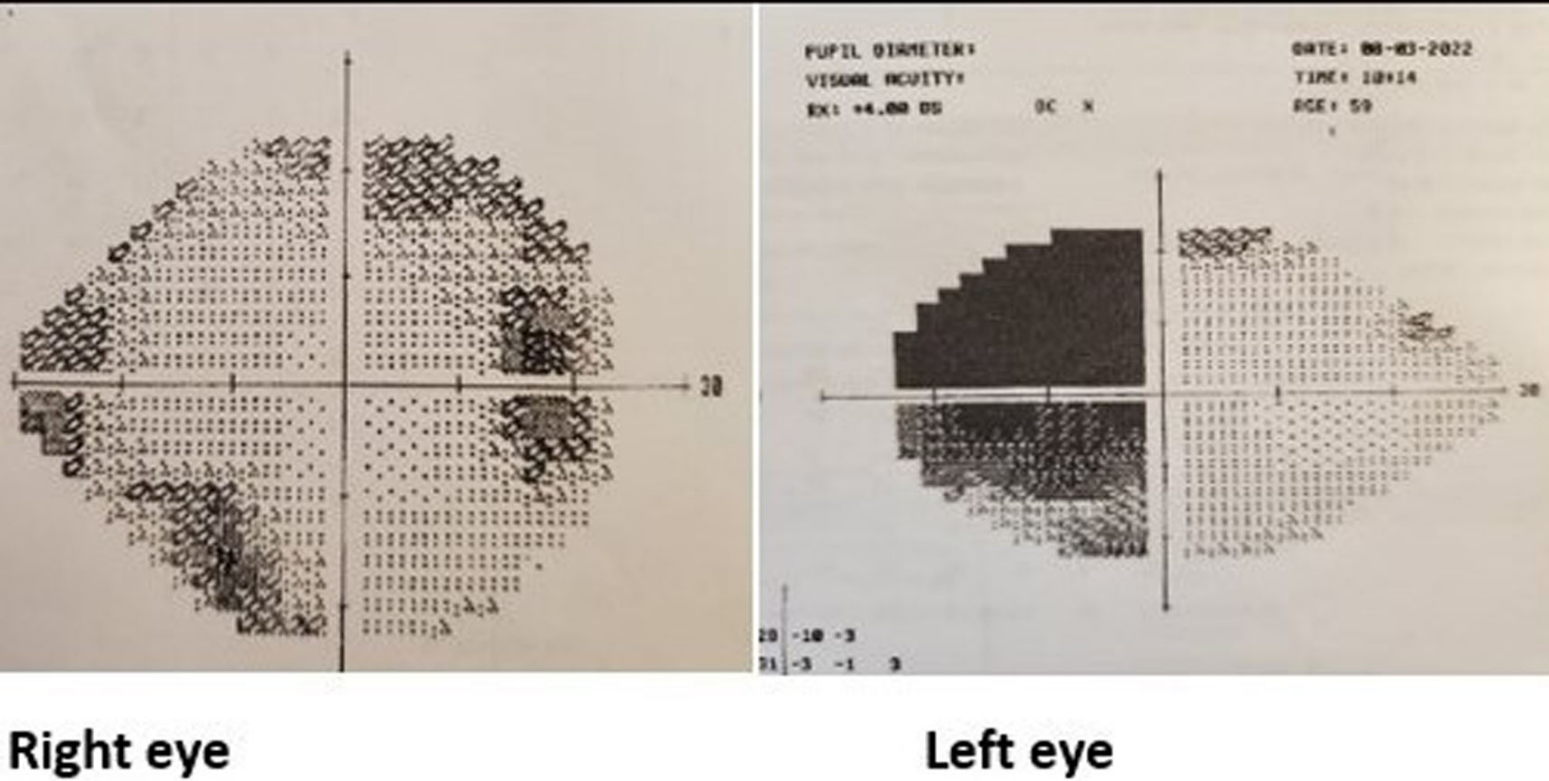
Visual field testing using Humphrey’s perimetry.

**Figure 2.**
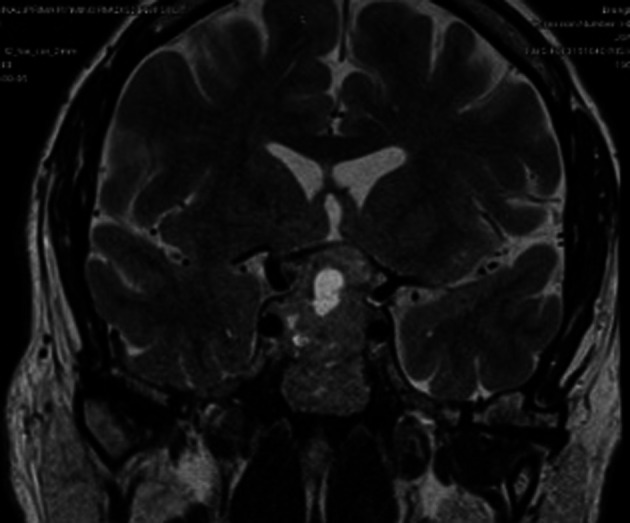
Large peripherally enhancing central and supra sellar necrotic mass measuring 33 × 29 × 26 mm.

His initial blood investigations ( Table 1) showed biochemical evidence of hypopituitarism with T4 of 9.5 pmol/L, FSH of 1.9 IU/L, LH of 2.0 IU/L, testosterone of 4.4 nmol/L, 9 am cortisol of 228 nmol/L and prolactin of 370 mU/L with a temporal field defect in the left eye, confirmed by Humphrey’s perimetry ( Figure 1). Pituitary MRI showed evidence of pituitary macroadenoma with suprasellar extension and optic chiasmal compression ( Figure 2).

**Table 1.** Baseline investigations.

Blood investigation	Value (normal range)
WBC	5.7 × 10 ^9^/L (4–11)
Hb	133 g/L (130–180)
PLT	240 × 10 ^9^/L (150–400)
Neu	4.1 × 10 ^9^/L (1.7–7.5)
Lymph	1.1 × 10 ^9^/L (1–4.5)
CRP	<5 mg/L (<5)
Glucose	5.7 mol/L (3–7.7)
TSH	0.9 mU/L (0.27–4.2)
T4	9.5 pmol/L (11–25)
Prolactin	370 mU/L (85–350)
Na	138 mmol/L (133–146)
K	4.8 mmol/L (3.5–5.3)
Urea	5.3 mmol/L (2.5–7.8)
Creatinine	82 umol/L umol/L (58–110)
eGFR	84 mL/min/1.73 m ^2^
Cortisol (9 am)	228 nmol/L (>420)
FSH	1.9 IU/L (1–12.0)
LH	2.0 IU/L (1.7–8.6)
Testosterone	4.4ng/dL (6.7–25.7)
IGF-1	11 mmol/L (5.9–27.3)

Abbreviations: WBC - White blood cell count, Hb - hemoglobin, PLT - platelet, Neu - neutrophil, Lymph-lymphocytes, CRP - C reactive protein, TSH - thyroid stimulating hormone, T4 - thyroxine, T3 - triiodothyronine, Na - sodium, K - potassium, eGFR - estimated glomerular filtration, FSH - follicular stimulating hormone, LH - luteinizing hormone, IGF-1 - insulin like growth factor 1.

The case was discussed among the multidisciplinary team for surgery. He was admitted after 3 months for transsphenoidal hypophysectomy. A complete excision was not possible because of the extent of the lesion. Immunohistological analysis showed gonadotropinoma with SF1 expression and negative FSH and LH beta subunits.

**Figure 3.**
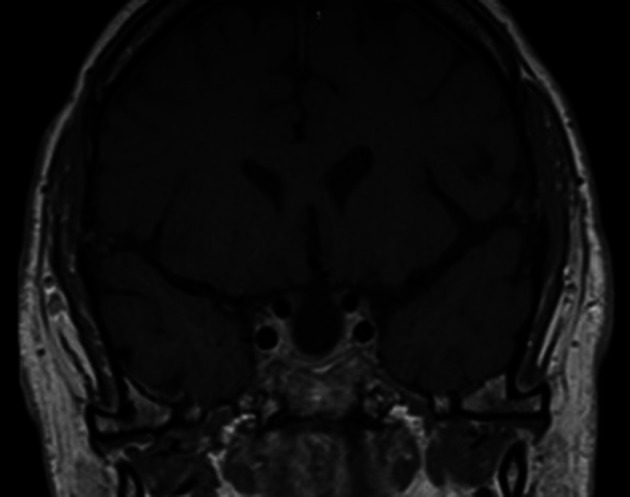
Postoperative follow-up magnetic resonance imaging after 3 months shows near complete resection of the tumour.

Mild postoperative hyponatremia was due to the syndrome of inappropriate antidiuretic hormone secretion (SIADH), which improved with fluid restriction. Perioperative intravenous hydrocortisone 50 mg 6 ^th^ hourly coverage was administered to prevent adrenal insufficiency. A low T4 level necessitated the administration of L-thyroxine (50 mcg once daily), but cortisol response was adequate and he was discharged with follow-up after 6 weeks. At follow-up, repeat anterior pituitary hormone profile test showed low TSH, T4, LH and testosterone levels, with a good response to the short synacthen test. Postoperative follow-up MRI after 3 months showed a near complete resection of the tumour ( Figure 3).

## Discussion

Non-functional pituitary adenomas (NFPAs) are increasingly common with a prevalence rate of 78–94 per 1000,000 population. ^1^ They are benign slow-growing tumours that arise from adenohypophyseal cells of the pituitary gland, with no clinical evidence of hormonal secretion. The most common NFPA is gonadrotropinoma, comprising approximately 80% of all NFPAs, expressing mainly LH, FSH, and alpha subunits along with other transcriptional factors such as SF1 and GATA2. ^2^ NFPAs are generally considered clinically silent tumours, but they can secrete small amounts of gonadotropins, rarely causing ovarian hyperstimulation in females and precocious puberty in males. ^3^ Our patient had SF1-positive immunohistochemistry, with patchy nuclear expression, negative FSH and LH subunits, with a Ki67 index of 2%, indicating a non-functional gonadotropinoma.

The patient was initially diagnosed with a visual field defect by an ophthalmologist and then was referred to an endocrinologist; detailed history taking showed a picture of gonadotropin deficiency. Patients with NFPA can present with acute deterioration in vision with or without headaches due to haemorrhage into the tumour (known as apoplexy), leading to rapid expansion of the tumour within the limited space of the sella turicia. ^4^ The patient had a left-sided visual field defect, with MRI showing a necrotic area within the tumour with no signs of bleeding or haemorrhage. Although bitemporal hemianopia is the most common type of visual field defect seen in 40% of patients, our patient had left superior and inferior temporal deficits without cranial nerve palsy. ^5^

Management includes screening of anterior pituitary hormones to rule out any functional secreting tumours such as prolactinoma. ^6^ Once deficiencies of two or more hormones are confirmed, the patient should be referred for visual field testing using either Humphrey’s or Goldmann perimetry. ^7^ Pituitary MRI with gadolinium contrast is recommended to identify the pituitary mass. ^8^ Transsphenoidal surgery is the treatment of choice for NFPA, and recovery depends on the tumour size. ^9^ Patients should have adequate perioperative hydrocortisone coverage to prevent adrenal insufficiency along with preoperative thyroid evaluation. ^9^ Transient complications such as diabetes insipidus or SIADH may occur postoperatively, from which patients may recover. ^10^ Our patient developed transient SIADH postoperatively on day 1 and recovered on day 4. Patients should be reassessed after 6 weeks with follow-up testing of the hypothalamo–pituitary–adrenocortical axis and MRI after 3 months; depending on the size of the lesion; 6 months to yearly surveillance may be required thereafter. ^9^

Some distinctive features to point out in our patient were firstly, it’s a Giant pituitary tumour with central necrotic area, resembling radiological features of pituitary apoplexy, which is a life-threatening condition, presented without headaches or vomiting. Secondly, the tumour was large enough to compress the optic chiasm but had affected the left temporal field sparing the right. Limitations to the study are that, since it is a single case study, the clinical features may vary and validation of our findings to a wider population is needed.

In conclusion, NFPA can remain asymptomatic for years and may present as visual disturbance, as seen in our patient. Most NFPAs are referred by ophthalmologists due to visual field defects on routine eye examinations. Large NFPAs with optic chiasmal compression need urgent referral for surgery.

## Consent

Written informed consent for their clinical details and clinical images was obtained from the patient.

## Data Availability

All data underlying the results are available as part of the article and no additional source data are required. Zenodo: CARE checklist for ‘Case Report: Atypical presentation of non-functional gonadotropinoma’, https://doi.org/10.5281/zenodo.7927437. ^11^ Data are available under the terms of the Creative Commons Attribution 4.0 International license (CC-BY 4.0).
