# Supplementary material for: Case Report: Atypical presentation of non-functional gonadotropinoma
Source: F1000Res. 2023 Sep 14;12:674. Originally published 2023 Jun 14. [Version 2] doi: 10.12688/f1000research.133438.2 (PMC10483175; doi:10.12688/f1000research.133438.2)
Supplement: Peer review history

# Reviewer report: Reviewer response for version 2

**DOI:** 10.5256/f1000research.155757.r206553

**Published:** 2023-09-20

**Authors:** Robinson George

**Affiliations:** 1 Department of General Surgery, Pushpagiri Institute of Medical Sciences and Research Centre, Tiruvalla, Kerala, India

Approved for indexing.

Are enough details provided of any physical examination and diagnostic tests, treatment given and outcomes?

Yes

Is the case presented with sufficient detail to be useful for other practitioners?

Yes

Is sufficient discussion included of the importance of the findings and their relevance to future understanding of disease processes, diagnosis or treatment?

Yes

Is the background of the case’s history and progression described in sufficient detail?

Yes

Reviewer Expertise:

My area of expertise is gastrointestinal & oncological laparoscopic surgery including endocrine breast.

I confirm that I have read this submission and believe that I have an appropriate level of expertise to confirm that it is of an acceptable scientific standard.

# Reviewer report: Reviewer response for version 2

**DOI:** 10.5256/f1000research.155757.r206552

**Published:** 2023-09-20

**Authors:** Atul Kalhan

**Affiliations:** 1 Department of Diabetes and Endocrinology, Royal Glamorgan Hospital, Llantrisant, UK

Approved.

Are enough details provided of any physical examination and diagnostic tests, treatment given and outcomes?

Yes

Is the case presented with sufficient detail to be useful for other practitioners?

Yes

Is sufficient discussion included of the importance of the findings and their relevance to future understanding of disease processes, diagnosis or treatment?

Yes

Is the background of the case’s history and progression described in sufficient detail?

Yes

Reviewer Expertise:

Endocrinology

I confirm that I have read this submission and believe that I have an appropriate level of expertise to confirm that it is of an acceptable scientific standard.

# Reviewer report: Reviewer response for version 1

**DOI:** 10.5256/f1000research.146428.r195186

**Published:** 2023-09-06

**Authors:** Atul Kalhan

**Affiliations:** 1 Department of Diabetes and Endocrinology, Royal Glamorgan Hospital, Llantrisant, UK

A well written case report related to a man who presented with left sided visual disturbance secondary to optic chiasm compression by a non-functioning macroadenoma (immunohistochemistry confirming it to be a gonadotropinoma). Patient had secondary hypogonadism and secondary hypothyroidism on presentation. It would be nice if a table with 6 weeks post-operative endocrine investigations are added as a comparison. 

 The last paragraph of the case report mentions about L-thyroxine (50 mcg once a day) being given on follow up. Was the patient put on long term thyroxine therapy (post-operatively)? It will be worth mentioning in the case report that thyroxine therapy should only be initiated if ACTH-cortisol axis integrity has been tested (prior to initiation of thyroxine therapy). A significant proportion of non-functioning pituitary adenomas show positive immunostaining for gonadotropins although the amount of gonadotropin secreted by such tumours is extremely small (to lead to a clinical impact) or it is a biologically inert gonadotropin. 

 In the discussion section, paragraph 3 has a statement which is factually incorrect and needs to be changed: Formal visual field assessment needs to be carried out for pituitary macroadenoma especially if these are in proximity of optic chiasm (or compressing the chiasm) irrespective of presence or absence of anterior pituitary hormonal deficiency. I will suggest the authors to change the 2nd line in this paragraph to reflect the above mentioned statement. In addition, the protocol for peri-operative coverage with hydrocortisone varies based on the institute (which is carrying out neurosurgery). I will suggest that the authors go through the following article by Prete *et al.* published in 2017 for reference (Prete *et al.*, 2017 ^1^).

Are enough details provided of any physical examination and diagnostic tests, treatment given and outcomes?

Yes

Is the case presented with sufficient detail to be useful for other practitioners?

Yes

Is sufficient discussion included of the importance of the findings and their relevance to future understanding of disease processes, diagnosis or treatment?

Yes

Is the background of the case’s history and progression described in sufficient detail?

Yes

Reviewer Expertise:

Endocrinology

I confirm that I have read this submission and believe that I have an appropriate level of expertise to confirm that it is of an acceptable scientific standard.

## References

[1] : Current best practice in the management of patients after pituitary surgery. *Ther Adv Endocrinol Metab* .2017;8(3) : 10.1177/2042018816687240 33-48 10.1177/2042018816687240 28377801PMC5363454

# Reviewer report: Reviewer response for version 1

**DOI:** 10.5256/f1000research.146428.r181637

**Published:** 2023-07-14

**Authors:** Robinson George

**Affiliations:** 1 Department of General Surgery, Pushpagiri Institute of Medical Sciences and Research Centre, Tiruvalla, Kerala, India

- This case of a non-functional pituitary adenoma has been well written with crisp history taking, precise examination findings & relevant investigations performed. 
- The case has been described well enough for the benefit of other general practitioners. 
- The case report has discussed the relevant investigations required & the precise management for the same.
- As a case report, it fulfills the concise format of information provided to the readers.
- Please check your spellings (sella turcica).

Are enough details provided of any physical examination and diagnostic tests, treatment given and outcomes?

Yes

Is the case presented with sufficient detail to be useful for other practitioners?

Yes

Is sufficient discussion included of the importance of the findings and their relevance to future understanding of disease processes, diagnosis or treatment?

Yes

Is the background of the case’s history and progression described in sufficient detail?

Yes

Reviewer Expertise:

My area of expertise is gastrointestinal & oncological laparoscopic surgery including endocrine breast.

I confirm that I have read this submission and believe that I have an appropriate level of expertise to confirm that it is of an acceptable scientific standard.
